# Supramolecular Linear-Dendritic Nanoreactors: Synthesis and Catalytic Activity in “Green” Suzuki-Miyaura Reactions

**DOI:** 10.3390/polym15071671

**Published:** 2023-03-28

**Authors:** Xin Liu, F. Max Yavitt, Ivan Gitsov

**Affiliations:** 1 Department of Chemistry, State University of New York—ESF, Syracuse, NY 13210, USA; 2 State Grid Corporation Joint Laboratory of Advanced Electrical Engineering Materials (SDEPC), State Grid Shandong Electric Power Research Institute, Jinan 250001, China; 3 Department of Chemical and Biological Engineering, University of Colorado Boulder, Boulder, CO 80303, USA; 4 The Michael M. Szwarc Polymer Research Institute, State University of New York, Syracuse, NY 13210, USA

**Keywords:** linear-dendritic, block copolymer, self-assembly, “green” chemistry, Suzuki coupling

## Abstract

This study describes the synthesis of novel amphiphilic linear-dendritic block copolymers and their self-assembly in water to form supramolecular nanoreactors capable of catalyzing Suzuki-Miyaura coupling reactions under “green” conditions. The block copolymers were formed through copper(I)-catalyzed alkyne-azide cycloaddition between azide functionalized poly(benzyl ether) dendrons as the perfectly branched blocks, as well as bis-alkyne modified poly(ethylene glycol), PEG, as the linear block. A first-generation poly(benzyl ether) dendron (G1) was coupled to a bis-alkyne modified PEG with molecular mass of 5 kDa, forming an ABA copolymer (G1)_2_-PEG5k-(G1)_2_ (yield 62%), while a second-generation dendron (G2) was coupled to a 11 kDa bis-alkyne modified PEG to produce (G2)_2_-PEG11k-(G2)_2_ (yield 49%). The structural purity and low dispersity of the linear-dendritic copolymers were verified by size-exclusion chromatography and matrix-assisted laser desorption/ionization time-of-flight mass spectrometry. Their self-assembly was studied by dynamic light scattering, showing that (G1)_2_-PEG5k-(G1)_2_ and (G2)_2_-PEG11k-(G2)_2_ formed single populations of micelles (17 nm and 37 nm in diameter, respectively). The triazole rings located at the boundaries between the core and the corona are efficient chelating groups for transition metals. The ability of the micelles to complex Pd was confirmed by ^1^H NMR, transmission electron microscopy, and inductively coupled plasma. The catalytic activity of the supramolecular linear-dendritic/Pd complexes was tested in water by model Suzuki-Miyaura reactions in which quantitative yields were achieved within 3 h at 40 °C, while, at 17 °C, a yield of more than 70% was attained after 17 h.

## 1. Introduction

Amphiphilic linear-dendritic copolymers are stimuli-responsive hybid macromolecules that self-assemble in different nanostructures depending on the surronding block-selective media [1,2,3,4,5]. Since their early inception, several publications have reported their ability to accommodate significant amounts of small molecules that are insoluble in the solvent chosen [6] or surface coat sizeable glycoproteins [7]. These properties facilitated the use of linear-dendritic block copolymers as efficient nanoreactors for “green” Diels Alder reactions between fullerene and polyaromatic hydrocarbons [8] and the first oxidation of fullerene mediated by an enzyme [9]. It should be emphasized that these unprecedented reactions were performed in aqueous media at ambient or close to ambient temperatures. Based on previous results, this study aims at the design and formation of novel linear-dendritic block copolymers and their evaluation as catalytic nanoreactors for another synthetically important process—the Suzuki-Miyaura (SM) reaction [10]. This method involves palladium-catalyzed cross-coupling of aryl bromide and boronic acid derivatives to yield a biaryl species. SM coupling is increasingly important in various applications, such as natural product synthesis [11], pharmaceuticals [12], and catalysis [13]. The process has many advantages: organoboron compounds are commercially available and environmentally safer than reactants used in other organic reactions. They are also easy to separate from reactions mixtures [14].

Despite the advantages of SM coupling, there is still a need for improvement. The reaction is normally conducted at high temperature in organic solvents [15], and thus is not environmentally friendly. In a world with increasing emphasis on environmental preservation, there is an interest to improve the process footprint by conducting this reaction in an aqueous media at milder temperatures [16]. Several attempts have been reported involving surfactants [17] and specifically designed micelles [18]. The elegant studies by Lipshutz’ group [18] have demonstrated the viability of this concept and were further extended by activation techniques [19] and dendrimers [20]. While there were previous publications of palladium encapsulation within the interior of the dendrimers [21], in most recent reports the catalyst was bound on complexation sites at their periphery [22] or adjacent to their periphery [23], facilitating in this way a favorable contact with the SM reagents involved. However, one problem with conducting the SM reaction in water is the low solubility of a wide range of interesting, but hydrophobic reactants. Therefore, it would be useful to develop a catalytic system that is soluble in water and can bind and solubilize hydrophobic substrates.

As was already mentioned, amphiphilic linear-dendritic copolymers form micelles in water and are capable of binding large amounts of hydrophobic substrates [6,8,9]. To improve on these previously reported linear-dendritic copolymer systems, in this study we have introduced a catalytic binding site at the junction between the linear hydrophilic block as the solubilizing entity and the hydrophobic dendritic “wedge” as the reagents reservoir. Triazole rings, formed throughout the dendrimer structure by copper(I)-catalyzed alkyne-azide cycloaddition (CuAAC), have been shown to bind Pd that catalyzes SM coupling reactions [24]. By introducing triazole rings into a linear-dendritic copolymer system, we aim to couple the catalytic ability of Pd with the high binding capacity and water solubility of the copolymer. In this manner, we hope to create a micellar catalytic system capable of acting as a nanoreactor for SM coupling reactions for hydrophobic reagents in an aqueous environment and under mild conditions.

## 2. Materials and Methods

### 2.1. Materials

All reagents and solvents were used without additional purification unless specified otherwise. Poly(ethylene glycol) with molecular mass 5000 Da (PEG5k, M_w_/M_n_ = 1.02) and 11,000 Da (PEG11k, M_w_/M_n_ = 1.04) were purchased from Polysciences, Inc. (Warrington, PA, USA). 2,2-Bis(hydroxymethyl)propionic acid, bis-MPA, (99+%) sodium azide, and NaN_3_ (98%, powder) were acquired from Acros. Sodium hydride, NaH, (95%, dry powder), 4-di(methylamino)pyridine, DMAP, (≥99%), copper(I) bromide, CuBr, (98%), propargyl bromide (80 wt.% in toluene), N,N′-dicyclohexylcarbodiimide, DCC, (99%), methyl 3,5-dihydroxybenzoate, 3,5-MDHB, (97%), 3,5-dihydroxybenzyl alcohol, 3,5-DHBA (98%), benzyl bromide (98%), sodium ascorbate, Na-Asc (≥99%), potassium carbonate, K_2_CO_3_ (≥98%, powder), 18-crown-6 (99%), 2,5-dihydroxybenzoic acid, 2,5-DHBA, (98%), phenylboronic acid, PBA, (95%), 4′-bromoacetophenone, 4′-BAcP, (99%), triethylamine, TEA (99.5%), and tetrahydrofuran, THF (HPLC grade) were all purchased from Sigma-Aldrich (Miwaukee, WI, USA). Dichloro-bis(benzonitrile) palladium, Pd(PhCN)_2_Cl_2_, (99%) was obtained from Strem Chemicals (Newburyport, MA, USA); diethyl ether, ethyl acetate, EtOAc, hexanes, and methanol (all reagent grade) were received from Pharmco, Inc. (Brookfield, CT, USA), and dichloromethane, DCM, (spectrophotometric grade) was purchased from Spectrum/Gleason Chemicals (Syracuse, NY, USA).

### 2.2. Instrumentation

#### 2.2.1. Size Exclusion Chromatography (SEC)

The analyses were completed on a line with M510 pump, U6K universal injector (both from Waters Co. Milford, MA, USA), three 5 μm PL Gel columns (50 Å, 500 Å and Mixed C, Agilent Tecgnologies, Inc. Santa Clara, CA, USA), and a Viscotek 250 dual refractive index/viscometry detector (Malvern Panalytical Inc. Westborough, MA, USA). The separation was achieved at 40 °C with THF (freshly distilled over KOH) eluting at 1 mL/min. The apparent molecular masses and molecular mass distributions were determined using 15 monodisperse poly(styrene) standards (162 Da–200 kDa, Polymer Standards Service, Warwick, RI, USA) and Viscotek OmniSEC software ver. 5.0.

#### 2.2.2. Matrix-Assisted Laser Desorption/Ionization—Time of Flight (MALDI-TOF)

The MALDI-TOF measurements were made on a Bruker Autoflex III MALDI-TOF instrument (Bruker Co. Billerica, MA, USA) with Smartbeam ion source equipped with the Nd-YAG laser (266, 355 nm). All spectra were recorded in a reflect-positive mode. A matrix was prepared by dissolving recrystallized 2,5-DHBA in methanol at a concentration of 40 mg/mL. The samples were prepared in methanol at a concentration of 1 mg/mL. Samples were spotted using the dried-droplet method with sample and matrix being premixed at a ratio of 1:7. An amount of 1 μL of mixed solution was spotted on AnchorChip target plate (MTP 384 polished steel, Bruker Daltonics).

#### 2.2.3. Nuclear Magnetic Resonance (NMR)

^1^H NMR spectra were recorded at room temperature on a Bruker AVANCE 600 MHz instrument (Bruker Co.) using 64 scanns and CDCl_3_ as the solvent and internal standard.

#### 2.2.4. Fluorescence Spectroscopy

Analyses were performed on a Horiba Fluorolog-3 spectrofluorometer (FL3-12, Horiba Jobin Yvon Inc. Edison, NJ, USA) equipped with a 450 W Xenon arc lamp. The emission spectra of polymer solutions ranging from 350 to 500 nm were recorded in a 10 mm quartz cuvette at an excitation wavelength of 334 nm and 1 mm slit width.

#### 2.2.5. Dynamic Light Scattering (DLS)

The DLS measurements were carried out on a Malvern Zetasizer ZS instrument (Malvern Panalytical Inc.). The instrument was equipped with a 633 nm laser source and a backscattering detector at 173°. Data were analyzed using a CONTIN procedure.

#### 2.2.6. Transmission Electron Microscopy (TEM)

TEM analyses were performed on JEOL JEM-2100F microscope (JEOL, Tokyo, Japan) at an accelerating voltage of 200 kV. A drop of sample solution at a concentration of 0.5–1 mg/mL was placed on a carbon-coated 400 square mesh copper grid. The solution excess was blotted with a lint-free filter paper after 1 min, and the grid was allowed to dry at ambient temperature for 1 h, and then it was vacuum-dried for 24 h.

#### 2.2.7. Inductively Coupled Plasma (ICP)

The palladium concentration in solution was determined on a Perkin Elmer Elan DRC-e mass spectrometer (Perkin Elmer Co., Hopkinton, MA, USA).

### 2.3. Methods

#### 2.3.1. Synthesis of 2,2-Bis(Propargyl) Propionic Acid (Bis-PPA)

This compound was synthesized from bis-MPA and propargyl bromide following a previously published procedure (Scheme S1) [24]. Total yield: 32%.

#### 2.3.2. Synthesis of Bis-Alkyne PEGs

The general procedure is described as follows: bis-alkyne functionalized PEGs were produced by coupling bis-PPA to PEG5k or 11k mediated by DCC. Predetermined quantities of PEG, DMAP, and bis-PPA were placed in a round bottom flask and dissolved in DCM. DCC was then added, and the solution was set to stir at room temperature for 18 h. The process was followed by thin layer chromatography (TLC). Upon completion, the reaction mixture was vacuum filtered to remove solids. The filtrate was precipitated drop wise in 60 mL of diethyl ether. Following precipitation, 20 mL more of diethyl ether were added, and the precipitate was set aside to aggregate for 20 min. The white solid was collected by vacuum filtration.

Bis-alkyne-PEG5k. PEG5k: 200 mg, bis-PPA: 174 mg, DCM: 2 mL, DCC: 160 mg, DMAP: 17 mg. Yield: 181 mg (84%).

^1^H NMR (600 MHz, CDCl_3_) δ 4.29 (t, *J* = 8.41 Hz, 4H), 4.17 (s, 8H), 3.78 (s, 8H), 3.67 (m, 440H), 2.46 (s, 4H), 1.25 (s, 6H).

Bis-alkyne-PEG11k. PEG11k: 300 mg, bis-PPA: 130 mg, DCM: 3 mL, DCC: 160 mg, DMAP: 16 mg. Yield: 245 mg (79%).

^1^H NMR (600 MHz, CDCl_3_) δ 4.20 (t, *J* = 8.27 Hz, 4H), 4.15 (s, 8H), 3.68 (s, 8H), 3.54 (m, 1000H), 2.32 (s, 4H), 1.28 (s, 6H).

#### 2.3.3. Synthesis of First-Generation Poly(Benzyl Ether) Dendron Bromide (G1-Br)

G1-Br was prepared from benzyl bromide and methyl 3,5-MDHB following previously published procedures, Scheme S2 [25,26]. Total yield: 74%.

#### 2.3.4. Synthesis of Second-Generation Poly(Benzyl Ether) Dendron Bromide (G2-Br)

G2-Br was synthesized from G1-Br and 3,5-DHBA using the original Hawker-Fréchet protocol [27]. Total yield: 19%.

#### 2.3.5. Synthesis of G1-N_3_ and G2-N_3_

The dendron-azides were formed by the following general procedure: G1-Br or G2-Br were dissolved in DMSO, and NaN_3_ was added, and the reaction was allowed to proceed at room temperature with TLC monitoring. Upon completion, the reaction mixture was extracted with three DCM portions. The solvent was evaporated, and the product was purified by column chromatography using hexanes (DCM (2:1, *v*/*v*)) as eluent mixtures. The solvents of the collected fractions wre removed to yield oily products.

G1-N_3_. G1-Br: 49 mg, NaN_3_: 57 mg, DMSO: 2 mL, DCM: 3 × 10 mL. Yield: 41 mg (93%).

G2-N_3_. G2-Br: 102 mg, NaN_3_: 58 mg, DMSO: 3 mL, DCM: 3 × 10 mL. Yield: 79 mg (81%).

#### 2.3.6. Synthesis of (G1)_2_-PEG5k-(G1)_2_, (G2)_2_-PEG5k-(G2)_2_, (G2)_2_-PEG11k-(G2)_2_

The linear-dendritic block copolymers were synthesized by the following unified method: A bis-alkyne-PEG and Gx-N_3_ were placed in a round-bottomed flask and dissolved in 1 mL acetone. CuSO_4_ solution in water was then added to this flask, along with sodium ascorbate. The mixture was stirred for 48 h at room temperature. At that time, the reaction mixture was extracted with three 10 mL DCM portions. The organic fractions were collected, and Na_2_SO_4_ was added to eliminate water traces. The resulting mixture was gravity filtered to remove the salt. The filtrate was concentrated by rotary evaporation, dissolved in DCM, and purified by column chromatography using increasing ratios of MeOH in DCM as eluent mixture.

(G1)_2_-PEG5k-(G1)_2_. G1-N_3_: 52 mg, bis-alkyne PEG5k: 71 mg, CuSO_4_: 6.3 mg, water: 0.5 mL, sodium ascorbate: 10.7 mg. Yield: 55 mg (63%).

^1^H NMR (600 MHz, CDCl_3_) δ 7.63 (s, 4H), 7.5–7.3 (m, 40H), 6.6–6.2 (m, 12H), 5.48 (s, 8H), 5.03 (s, 16H), 4.61 (s, 8H), 4.17 (t, *J* = 6.75 Hz, 4H), 3.77 (t, *J* = 7.2 Hz, 4H), 3.66 (m, 440H), 1.15 (s, 6H).

(G2)_2_-PEG5k-(G2)_2_. G2-N_3_: 44 mg, bis-alkyne PEG5k: 50 mg, CuSO_4_: 6.5 mg, water: 0.25 mL, sodium ascorbate: 10 mg. Yield: 47 mg (60%).

^1^H NMR (600 MHz, CDCl_3_) δ 7.69 (s, 4H), 7.38 (m, 80H), 6.66–6.5 (m, 36H), 5.39 (s, 8H), 5.0 (s, 32H), 4.92 (s, 16H), 4.65 (s, 8H), 4.11 (t, *J* = 6.9 Hz, 4H), 3.81 (t, *J* = 7.3 Hz, 4H), 3.6 (m, 440 H), 1.14 (s, 6H).

(G2)_2_-PEG11k-(G2)_2_. G2-N_3_: 120 mg, bis-alkyne PEG11k: 93 mg, CuSO_4_: 10 mg, water: 0.4 mL, sodium ascorbate: 14 mg. Yield: 58 mg (49%).

^1^H NMR (600 MHz, CDCl_3_) δ 7.72 (s, 4H), 7.4 (m, 80H), 6.64–6.52 (m, 36H), 5.44 (s, 8H), 5.01 (s, 32H), 4.9 (s, 16H), 4.62 (s, 8H), 4.17 (t, *J* = 6.8 Hz, 4H), 3.78 (t, *J* = 7.2 Hz, 4H), 3.66 (m, 1000 H), 1.15 (s, 6H).

#### 2.3.7. Critical Micelle Concentration (CMC)

The self-assembly onset of the linear-dendritic block copolymers was determined by fluorescence spectroscopy in deionized (DI) water (18.2 MΩ) with pyrene (99%, Aldrich) as the fluorescent probe. The onset of sharp increase in the third vibronic band intensity *I*_383_ was used to determine cmc [6]. CMC_(G1)2-PEG5k-(G1)2_ = 4.4 mg/mL (6.51 × 10^−4^ M); CMC_(G2)2-PEG11k-(G2)2_ = 2.9 mg/mL (2.55 × 10^−4^ M).

#### 2.3.8. Pd-Micelle Formation

(G1)_2_-PEG5k-(G1)_2_ stock solution in THF was made at a concentration of 4.4 mg/mL. Appropriate volume (230 μL) of the stock solution was added to a small vial so that the vial contained 1 mg of the copolymer (1.478 × 10^−7^ M) and the solvent was evaporated. Fourfold excess (2 eq per binding site) of PdCl_2_(PhCN)_2_—227 mg (5.914 × 10^−7^ M) was dissolved in 5 mL of THF, and 50 μL of that solution was added to the vial containing the (G1)_2_-PEG5k-(G1)_2_, producing a slightly yellowish clear solution. The vial was capped over night to prevent the THF from evaporating. An amount of 100 μL from this solution was slowly injected into 1 mL of water, and THF was allowed to evaporate under air flow. The resulting clear aqueous solution was then analyzed by DLS, TEM, and ICP. ICP revealed that the solution contained 0.567 mg Pd, or 90% of the theoretical value (assuming 2 eq of PdCl_2_ can be bound per 1 molecule of (G1)_2_-PEG5k-(G1)_2_).

#### 2.3.9. Suzuki-Miyaura Catalysis

Phenylboronic acid (2.5 mg, 2.05 × 10^−5^ M), 4′-bromoacetophenone (1.99 mg, 1 × 10^−5^ M) and triethylamine, TEA (4.14 μL, 3 mg, 2.97 × 10^−5^ M), were added to a small round bottom flask. An amount of 1 mL of (G1)_2_-PEG5k-(G1)_2_ water solution with 90% Pd (0.567 mg) was added to the flask. The reaction mixture was stirred at 40 °C for 3 h and was then extracted three times with 3 mL diethyl ether. The organic fractions were collected and dried over Na_2_SO_4_. The clear supernatant was decanted, and, after solvent evaporation, a white solid was obtained. Yield: 1.95 mg (99.5%). The product was dissolved in deuterated chloroform and analyzed by NMR.

^1^H NMR (600 MHz, CDCl_3_) δ 8.04 (d, *J* = 8.27 Hz, 2H), 7.69 (d, *J* = 8.22 Hz, 2H), 7.63 (d, *J* = 7.54 Hz, 2H), 7.48 (t, *J* = 7.83 Hz, 2H), 7.41 (t, *J* = 7.53 Hz, 1H), 2.63 (s, 3H), Figure S1B.

## 3. Results and Discussion

### 3.1. Synthesis of Linear Dendritic Block Copolymers

**Scheme 1:**
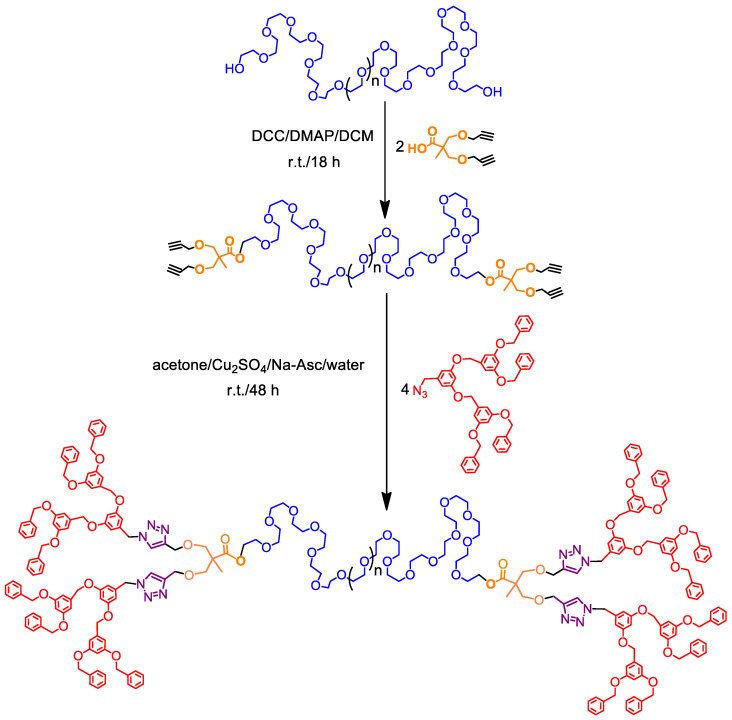
Synthesis of (G2)_2_−PEG−(G2)_2_. PEG5k: n = 97; PEG 11k: n = 233.

**Figure 1:**
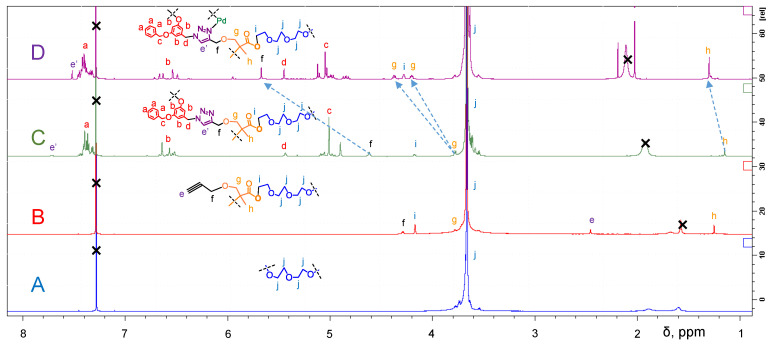
^1^H NMR spectra of PEG5k (**A**), bis-alkyne PEG5k (**B**), (G1)_2_−PEG5k−(G1)_2_ (**C**) and (G1)_2_−PEG5k−(G1)_2_/Pd complex (**D**). Blue labels: PEG protons, orange labels: bis-PPA protons, purple labels: methine/triazole protons, red labels: poly(benzyl ether) dendron protons. ×—solvent protons. See Section 2.2.3. in ‘Materials and Methods’ for analysis conditions.

**Figure 2:**
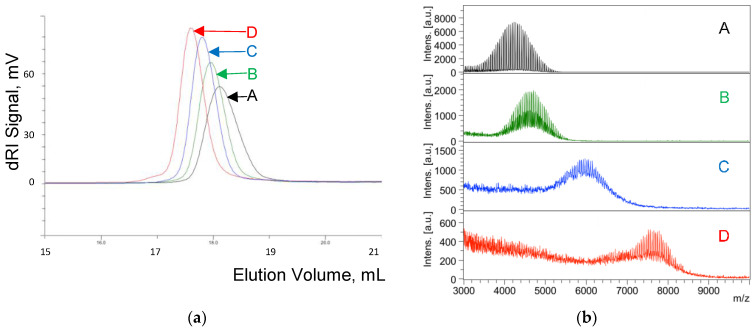
Analysis of starting material PEG 5k, A, intermediate (alkyne)-PEG5k-(alkyne), B, and final products (G1)_2_−PEG5k−(G1)_2_, C, (G2)_2_−PEG5k−(G2)_2_, D: (**a**) Overlay of SEC eluograms; (**b**) Overlay of MALDI-TOF spectra. See Section 2.2.1 and Section 2.2.2 in Materials and Methods for analysis conditions.

The synthetic sequence is shown in Scheme 1. Firstly, the bis-alkyne PEGs were formed by coupling of bis-PPA to PEG 5k (Scheme 1, n = 97) or PEG11k (Scheme 1, n = 233) catalyzed by DCC/DMAP. Following the coupling, the reaction mixture turned brown and produced a white solid after precipitation in diethyl ether. The yields were relatively high—84% with PEG5k and 79% with PEG11k. Signals typical for the addition of bis-PPA—4.29 ppm -OC*H*_2_C≡CH, 4.17 ppm -CH_2_C*H*_2_-OC(O)-, 3.78 ppm -C(CH_3_)-C*H*_2_-O-; 2.46 ppm -C≡C*H,* and 1.25 ppm -C-C*H*_3_—appeared along with the methylene PEG protons (3.67 ppm) in the ^1^H NMR spectra, Figure 1B. The desired linear-dendritic block copolymers were then obtained by a CuAAC “click” reaction between azide functionalized G1 (not shown) and G2 (Scheme 1) dendrons and bis-alkyne terminated PEGs. A total of three products were created: (G1)_2_-PEG5k-(G1)_2_, (G2)_2_-PEG5k-(G2)_2_, and (G2)_2_-PEG11k-(G2)_2_. At the start, the reaction mixtures were cloudy due to G1-N_3_ and G2-N_3_ insolubility in water, but they gradually cleared as the reaction progressed. The copolymer yields were as follows: (G1)_2_-PEG5k-(G1)_2_: 63%; (G2)_2_-PEG5k-(G2)_2_: 60%, and (G2)_2_-PEG11k-(G2)_2_: 49%. The starting PEGs, the intermediates, and the final products of each synthetic sequence were analyzed by ^1^H NMR (Figure 1C for PEG5k), as well as by SEC and MALDI -TOF, to confirm their structural purity. An example is shown in Figure 2.

The SEC overlay of the starting PEG5k, bis-alkyne PEG5k, (G1)_2_-PEG5k-(G1)_2_, and (G2)_2_-PEG5k-(G2)_2_ revealed unimodal peaks with no trace of the starting reagents (Figure 2a). The decreasing elution volume observed for each peak confirmed the increase in the hydrodynamic volume (i.e., the molecular mass) after each synthetic step, while the dispersity Ð of each polymer product remained narrow (Table S1).

The MALDI-TOF overlay, shown in Figure 2b, provided additional evidence for the structural purity. Upon addition of the small bis-alkyne moieties, all of the molecular peaks were shifted by approximately 400 Da, corresponding to the addition of two bis-alkyne groups (M = 210 Da each) to both PEG chain ends. Similarly, upon addition of four G1 dendrons (M = 345 Da each) to the PEG chains, the molecular ion peaks were seen to shift by ~1300 Da. Finally, the addition of four G2 dendrons (M = 769 Da each) caused the peaks of the bis-alkyne modified PEG to shift simultaneously by ~3000 Da. The increasing loss of resolution at higher molecular weights (Figure 2b, C and D) has been previously observed with other ethylene glycol macromolecules [24]. It should be mentioned, however, that both the SEC traces and the MALDI-TOF spectra showed no presence of the starting material after purification at each synthetic step. The ^1^H NMR spectra of the initial PEG5k, the intermediates, and the final product are shown in Figure 1A–C.

**Figure 3:**
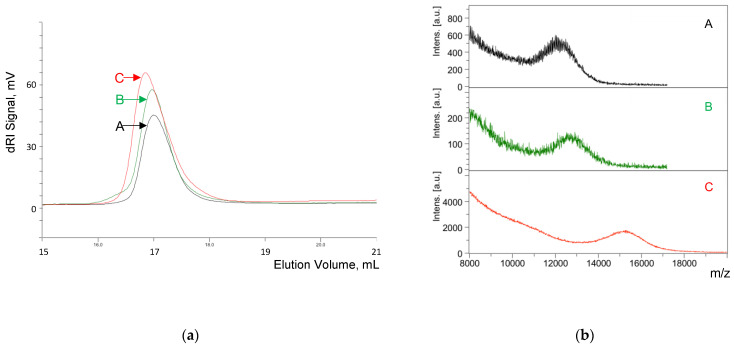
Analysis of starting material PEG11k, A, intermediate (alkyne)−PEG11k−(alkyne), B, and final product (G2)_2_−PEG11k−(G2)_2_, C: (**a**) Overlay of SEC eluograms; (**b**) Overlay of MALDI-TOF spectra. See Section 2.2.1 and Section 2.2.2 in Materials and Methods for analysis conditions.

In contrast to the PEG5k copolymers, the yield of (G2)_2_-PEG11k-(G2)_2_ was notably lower (49%). The low yield is likely due to a combination of incomplete coupling and loss of product during purification. After separation of the reaction products by column chromatography, several of the isolated fractions showed presence of unreacted (alkyne)-PEG11k-(alkyne), necessitating repeated fractionation. The likely reason for the separation difficulty might be the insignificant change in the molecular mass after the addition of the (G2) dendrons to the (alkyne)-PEG11k-(alkyne) in distinction to the PEG5k based block copolymers. The SEC and MALDI-TOF analyses are presented in Figure 3. Both overlays showed similar trends—each addition caused an increase in hydrodynamic volume (SEC, Figure 3a) and an increase in the values of molecular ion peaks (MALDI-TOF, Figure 3b). Again, traces of starting reagents were not observed after each synthetic step, while the loss of resultion in the MALDI-TOF spectra is even more pronounced due to the reasons already discussed. The molecular masses and molecular mass distributions of the PEG11k based products are summarized in Table S1.

### 3.2. Self-Assembly of Linear Dendritic Block Copolymers

**Figure 4:**
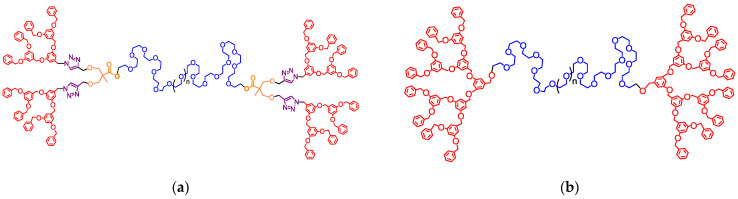
Structures of linear dendritic block copolymers containing the same amount of poly(benzyl ether) dendrons attached to the same PEG block (n = 233): (**a**) (G2)_2_−PEG11k−(G2)_2_, this work; (**b**) [G-3]−PEG11k−[G-3], [1,6].

**Figure 5:**
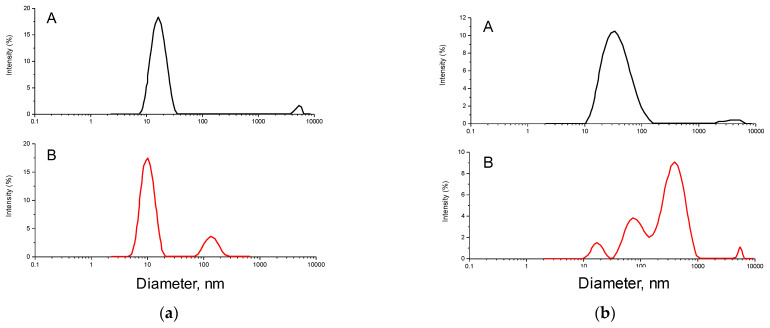
Dynamic light scattering of aqueous solutions before (A) and after addition of PdCl_2_(PhCN)_2_ (B): (**a**) (G1)_2_−PEG5k−(G1)_2_ micelles; (**b**) (G2)_2_−PEG11k−(G2)_2_ micelles. See Section 2.2.5, Section 2.3.7 and Section 2.3.8 in ‘Materials and Methods’ for analysis conditions.

(G1)_2_-PEG5k-(G1)_2_ and (G2)_2_-PEG11k-(G2)_2_ were soluble in water and self assembled to form micelles. Interestingly, they contained the same amount of poly(benzyl ether) dendrons attached to the same PEG block as those previously investigated (Figure 4), but they self-assembled at notably higher concentrations—(G1)_2_-PEG5k-(G1)_2_ CMC = 6.51 × 10^−4^ M vs. [G-2]-PEG5k-[G-2] CMC = 2.0 × 10^−5^ M, as well as (G2)_2_-PEG11k-(G2)_2_ CMC = 2.6 × 10^−4^ M vs. [G-3]-PEG11k[G-3] CMC = 7.1 × 10^−6^ M. [6] The difference was in the connecting unit in the two families: a more flexible bis-PPA (Figure 4a) vs. a more stiff 3,5-dihydroxy benzyl (Figure 4b). The flexibility of the bis-PPA enables better accommodation of the hydrophobic G1 and G2 dendrons in the micellar core, resulting in a single population of supramolecular assemblies with smaller sizes than the ones previously reported—17 nm vs. 32 nm for the PEG5k copolymers and 37 nm vs. 44 nm for the PEG 11k copolymers, Figure 5ab, A [6]. In analogy with previously synthesized [G-3]-PEG5k-[G-3] ([1]), the newly formed (G2)_2_-PEG5k-(G2)_2_ did not dissolve in water forming transparent physical networks, which were not further investigated.

**Figure 6:**
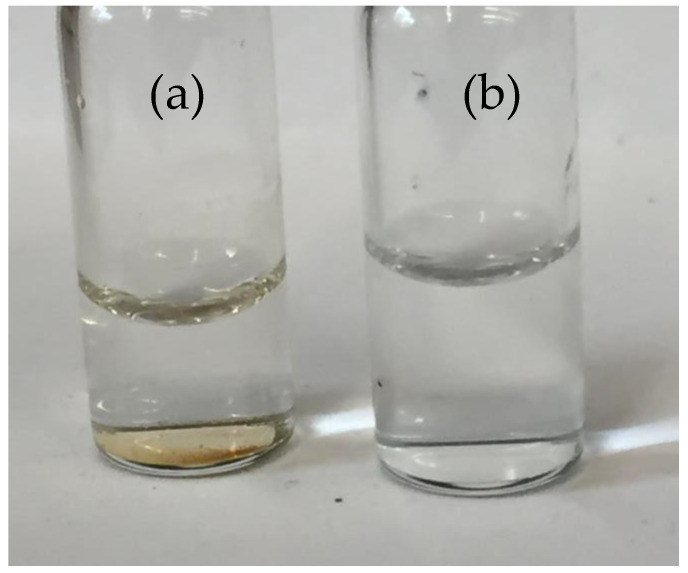
Aqueous solutions of PdCl_2_(PhCN)_2_: (**a**) without (G1)_2_−PEG5k−(G1)_2_ micelles; (**b**) with (G1)_2_−PEG5k−(G1)_2_ micelles.

**Figure 7:**
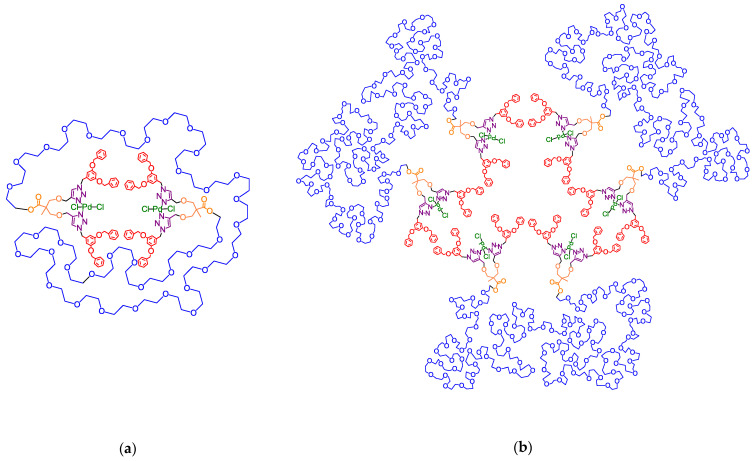
Schematic cross-section representation of Pd loaded micelles formed in water by solvent displacement: (**a**) Monomolecular (G1)_2_−PEG5k−(G1)_2_ micellar Pd complex; (**b**) Multimolecular (G1)_2_−PEG5k−(G1)_2_ micellar Pd complex.

It has been shown previously that triazole rings attached to bis-MPA dendritic branching are able to efficiently bind Pd salts [24]. A simple experiment was used to test the binding ability of the newly synthesized materials by adding bis(benzonitrile)palladium(II) chloride to water with and without the novel linear-dendritic block copolymers, Figure 6. In pure water, the Pd salt precipitated (orange solid on the bottom of the vial, Figure 6a), while the copolymer solution became clear after vortex stirring for 30 s, Figure 6b. The complexation of the palladium(II) chloride was manifested by distinct chemical shifts in the ^1^H NMR spectra, Figure 1D. The positions of the protons in the poly(benzyl ether) dendrons remained unchanged, but the protons associated with the bis-PPA moiety and triazole rings shifted, Figure 1C,D. The DLS scans of the linear dendritic copolymer micelles with molecularly bound PdCl_2_ differed from the pure linear-dendritic micelles, Figure 5a,b, B. The Pd-containing (G1)_2_-PEG5k-(G1)_2_ micelles existed as two species, one with a diameter of 10 nm and one with a diameter of 120 nm (Figure 5a, B). The (G2)_2_-PEG11k-(G2)_2_ micelles bound to Pd seemed to form three populations, with diameters of approximately 19, 90 and 300 nm (Figure 5b, B). The multiplicity of sizes observed after the addition of PdCl_2_(PhCN)_2_ indicated a different mechanism of self-assembly, which was seemingly influenced by the Pd complexation. In all probability, the fractions with the smallest sizes consisted of monomolecular micelles (at 1 mg/mL both copolymers concentrations were below cmc), while the large size fractions contained multimolecular micelles assembled through the formation of hydrophobic intramolecular Pd complexes. Both structures are shown in Figure 7.

**Figure 8:**
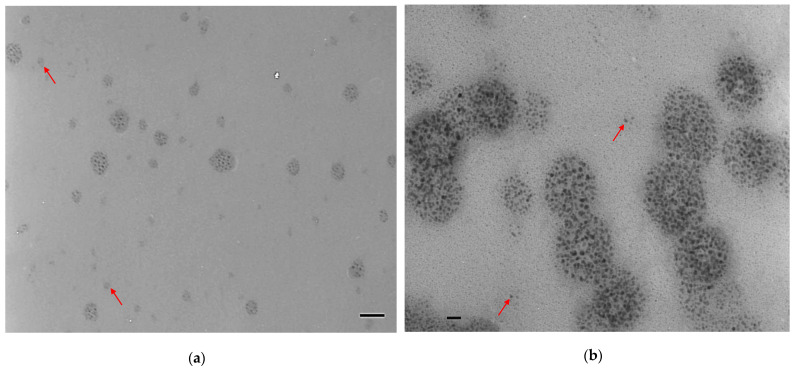
TEM micrographs of Pd-loaded linear dendritic block copolymers.: (**a**) (G1)_2_−PEG5k−(G1)_2_ micellar Pd complex; (**b**) (G2)_2_−PEG11k−(G2)_2_ micellar Pd complex. Scale bar: 50 nm. Red arrows mark possible Pd monomolecular micelle complexes. See Section 2.2.6 and Section 2.3.8 in ‘Materials and Methods’ for analysis conditions.

The DLS data were supported by the TEM analyses of both Pd-containig (G1)_2_-PEG5k-(G1)_2_ and (G2)_2_-PEG11k-(G2)_2_, as shown in Figure 8. The coexistence of particles with different dimensions seen in the DLS scans appeared also in the TEM images. The numerical difference between DLS and TEM sizes where larger structures seemed to appear should be attributed to the flattening of the micelles upon deposition on the TEM grid. The existence of interconected (G2)_2_-PEG11k-(G2)_2_ supermolecules was a notable difference frequently observed in the TEM images, Figure 8b. Most probably, the sufficiently long PEG11k chain enables the dendrons of some free floating linear-dendritic block copolymers to anchor in two or more neighboring micelles and “stitch” them together. A schematic representation of this process with TEM images of previously synthesized individual [G3]-PEG11k-[G3] micelles and their “stitched” derivatives are shown in Figure S1.

### 3.3. Evaluation of Catalytic Activity

**Scheme 2:**
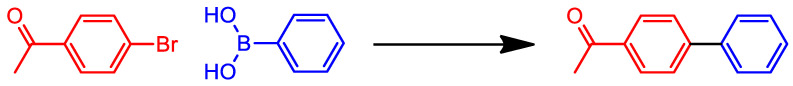
Synthesis of 4-acetylbiphenyl. 4′-BAcP:PBA:TEA (1:2:3 eq), water, 40 °C, 3 h.

The model Suzuki-Miyaura coupling reaction, shown in Scheme 2, was used to characterize the catalytic activity of the Pd linear-dendritic micelles. The reactants used in this reaction, 4′-bromoacetophenone and phenyl boronic acid, were chosen due to their hydrophobicity and ease of separation. The (G1)_2_-PEG5k-(G1)_2_ micelle was used because of its relatively better size uniformity. The reaction was performed in water at two sets of conditions: 40 °C/3 h and 17 °C/17 h. Following the time allocated for the catalysis, the reaction mixture was extracted in diethyl ether. The micelles were insoluble in ether, and thus remained in the aqueous phase, while the catalysis reactants and products were separated to the organic phase. The NMR spectra of the reaction mixture after 3 h at 40 °C (Figure S2B) showed no peaks corresponding to 4′-bromoacetophenone, the limiting reagent, and thus the catalysis was determined to produce 100% conversion (yield of isolated product 99.5%). The yield of pure product isolated after 17 h at 17 °C. was 72%. When (G2)_2_-PEG11k-(G2)_2_ Pd complex was used with identical reagent ratios and 17 °C/20 h, the yield of the isolated product was 62%. After the reaction was performed with pure PdCl_2_(PhCN)_2_ in water and the same reagent ratios, but without the copolymers, no 4-acetylbiphenyl could be isolated, with only traces visible in the ^1^H NMR spectrum.

## 4. Conclusions

The results obtained in this study showed that amphiphilic linear-dendritic block copolymers could be successfully produced by a “click” reaction between PEGs and poly(benzyl ether) dendrons with complimentary designed coupling sites. Compared with the previously reported Williamson ether syntheses using the same linear and dendritic blocks [1], the yields were lower. The two most probable reasons for the lower yields were (1) the steric hindrance experienced by the dendrons approaching the two alkyne groups on each bis-PPA moiety and (2) the rather complex mechanism of the CuAAC reaction proceeding through a sterically demanding intermediates [28]. However, what was lost in yield was compensated by a dual gain-of-function. Firstly, the insertion of the bis-PPA ester group between the linear and the dendritic blocks would enable the controllable split of the hydrophilic PEG corona from the hydrophobic dendritic core under acid-, base-, or enzymatic action, thus opening interesting avenues towards promising medical applications [29]. Secondly, the site-selective positioning of metal binding triazole pairs at the interface between the core and the corona of the linear-dendritic micelles (Figure 7) would facilitate the complexation of metals with biomedical or catalytic functions. The pilot experiments performed in this study indicated that Pd-loaded micelles made of (G1)_2_-PEG5k-(G1)_2_ were able to efficiently bind hydrophobic substrates and expedite their chemical transformation under the Suzuki-Miyaura mechanism. The advantageous complexation of the Pd catalyst at the interface between the core and the corona of the supramolecular formations (Figure 7) possibly enabled an intimate contact between the participating SM reagents. While the achieved ~100% yield at 40 °C was a laudable accomplishment, the yields at 17 °C were lower for both copolymers tested. There are still many ways to improve this system. Possibly greener conditions for the synthesis of the linear-dendritic block copolymers or their greener analogues could be found. Current experiments suggested that the micellar/Pd complex can be recovered after one catalytic reaction. Future trials will expand the range of aromatic halides, explore Pd complexation within (G2)_2_-PEG5k-(G2)_2_ physical networks, and investigate the catalytic activity of recycled catalysts (micelles and physical hydrogels).

## Data Availability

The experimental results presented in this study are contained within this article and the associated supplementary materials.

## Supplementary Materials

The following supporting information can be downloaded at: https://www.mdpi.com/article/10.3390/polym15071671/s1, Scheme S1: Synthesis of 2,2-bis(propargyl) propionic acid (bis-PPA); Scheme S2: Synthesis of first generation poly(benzyl ether) dendron azide; Table S1: Molecular mass characteristics of initial PEGs, intermediates and final products in the synthesis of linear-dendritic copolymers; Figure S1: Schematic representation of micelle “stitching” and TEM images of individual [G-3]-PEG11k-[G-3] micelles and their interconnected supermolecules; Figure S2: ^1^H NMR spectra of 4-bromoacetophenone (A) and S-M coupling product with phenyl boronic acid (B).
