# Supplementary material for: Supramolecular Linear-Dendritic Nanoreactors: Synthesis and Catalytic Activity in “Green” Suzuki-Miyaura Reactions
Source: Polymers (Basel). 2023 Mar 28;15(7):1671. doi: 10.3390/polym15071671 (PMC10096851; doi:10.3390/polym15071671)

Supporting Information

Supramolecular Linear-Dendritic Nanoreactors: Synthesis and Catalytic Activity in "Green" Suzuki-Miyaura Reactions

Xin Liu [1,2], Francis Max Yavitt [1,3] and Ivan Gitsov [1,4,*]

[1]Department of Chemistry, State University of New York - ESF, Syracuse, NY 13210; xliu70@syr.edu
[2]State Grid Corporation Joint Laboratory of Advanced Electrical Engineering Materials (SDEPC), State Grid Shandong Electric Power Research Institute, Jinan 250001, China
[3]Department of Chemical and Biological Engineering, University of Colorado Boulder, Boulder, CO 80303; francis.yavitt@colorado.edu
[4]The Michael M. Szwarc Polymer Research Institute, State University of New York, Syracuse, NY 13210; igivanov@syr.edu

*Correspondence: igivanov@syr.edu; Tel.: 1-315-470-6860

CONTENTS

Scheme S1: Synthesis of 2,2-bis(propargyl) propionic acid (bis-PPA)……….……….……….…...….S2

Scheme S2: Synthesis of first generation poly(benzyl ether) dendron azide....…..……….……......S2

Table S1: Molecular mass characteristics of initial PEGs, intermediates and final products in the synthesis of linear-dendritic copolymers………………...…………………………………….……S3

Figure S1: Schematic representation of micelle "stitching" and TEM images of individual [G-3]-PEG11k-[G-3] micelles and their interconnected supermolecules …………………………...…S4

Figure S2: ^{1}H NMR spectra of 4-bromoacetophenone (A) and S-M coupling product with phenyl boronic acid (B)……………………………………...…………………………………S5

Scheme S1: Synthesis of 2,2-bis(propargyl) propionic acid (bis-PPA)

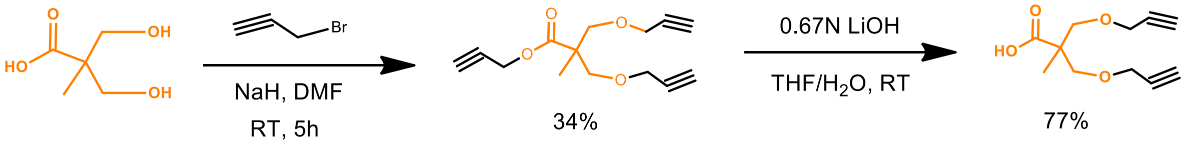

L. Wang, D.J. Kiemle, C.J. Boyle, E.L. Connors, I. Gitsov
"Click" Synthesis of Intrinsically Hydrophilic Dendrons and Dendrimers Containing Metal Binding Moieties at Each Branching Unit.
Macromolecules **47(6)**, 2199-2213 (2014)

Scheme S2: Synthesis of first generation poly(benzyl ether) dendron azide

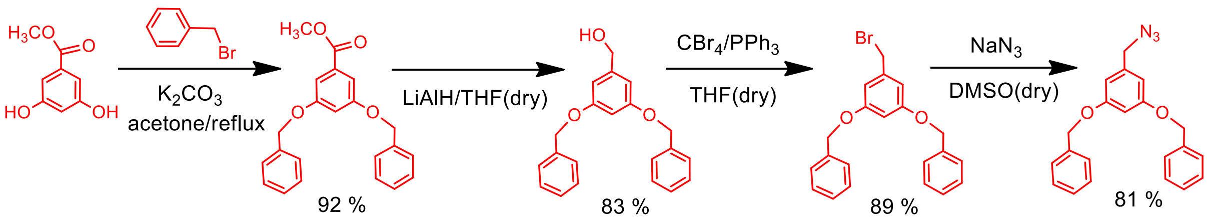

C. Hawker, J.M.J. Fréchet
A New Convergent Approach to Monodisperse Dendritic Macromolecules.
J. Chem. Soc., Chem. Commun. **1990**, 1010-1013.

Xin Liu, F. Max Yavitt, Ivan Gitsov
Supramolecular Linear-Dendritic Nanoreactors: Synthesis and Catalytic Activity in "Green" Suzuki-Miyaura Reactions
Polymers main text (2023)

Table S1: Molecular mass characteristics of initial PEGs, intermediates and final products in the synthesis of linear-dendritic copolymers

Compound	M_p[a)]	M_n[b)]	M_w[b)]	Đ[c)]
**PEG5k**	4210	4030	4140	1.03
(alkyne)-PEG5k-(alkyne)	4640	4500	4630	1.03
$(G1)_2$-PEG5k-$(G1)_2$	5150	4950	5100	1.10
$(G2)_2$-PEG5k-$(G2)_2$	6000	5710	6040	1.06
**PEG11k**	12200	9750	11800	1.21
(alkyne)-PEG11k-(alkyne)	12700	10600	13500	1.27
$(G2)_2$-PEG11k-$(G2)_2$	156000	11110	14150	1.27

[a)]Apparent molecular mass measured at the apex of the eluting peak, [b)]apparent molecular mass calculated against poly(styrene) calibration, [c)]polymer dispersity M_w/M_n.

Figure S1: Schematic representation of micelle "stitching" and TEM images of individual [G-3]-PEG11k-[G-3] micelles (a, scale bar 50 nm) and their interconnected supermolecules (b, scale bar 200 nm)

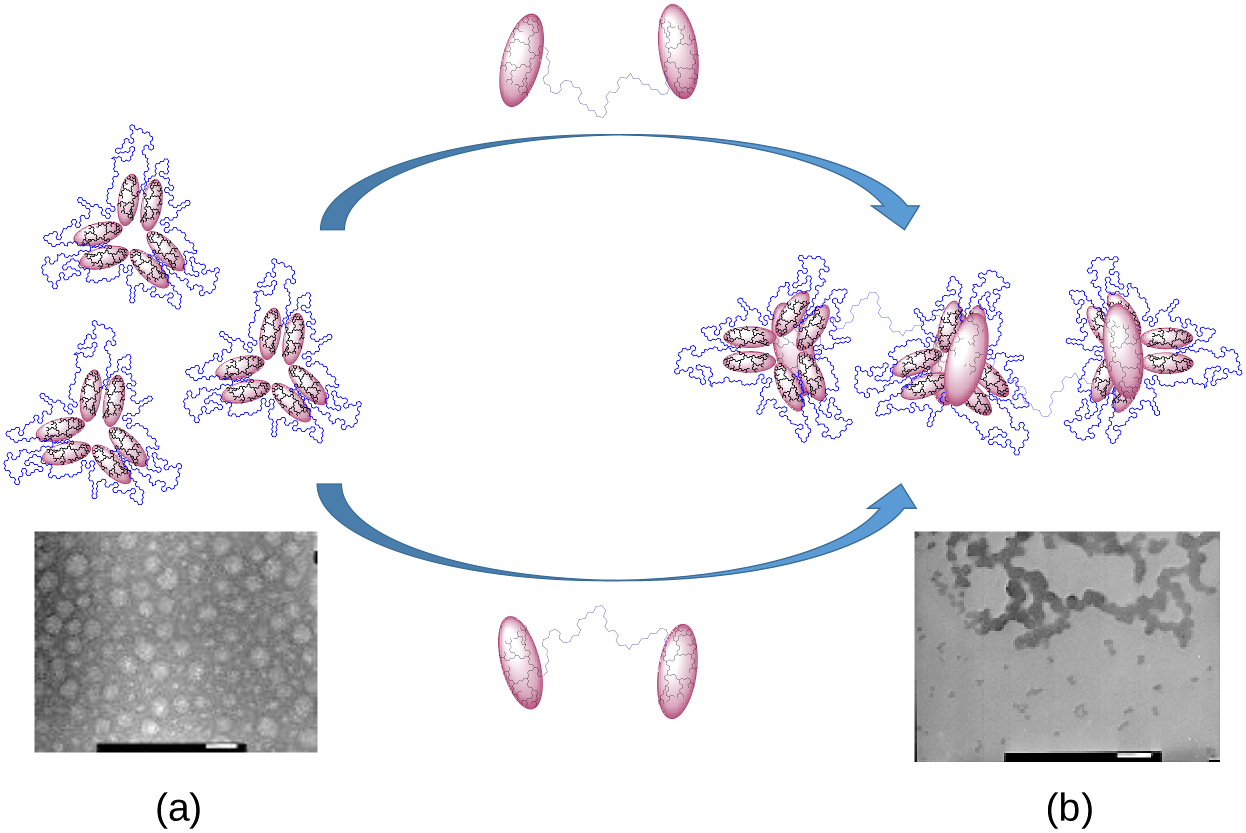

Figure S2: ^{1}H NMR spectra of 4-bromoacetophenone (A) and S-M coupling product with phenyl boronic acid (B)

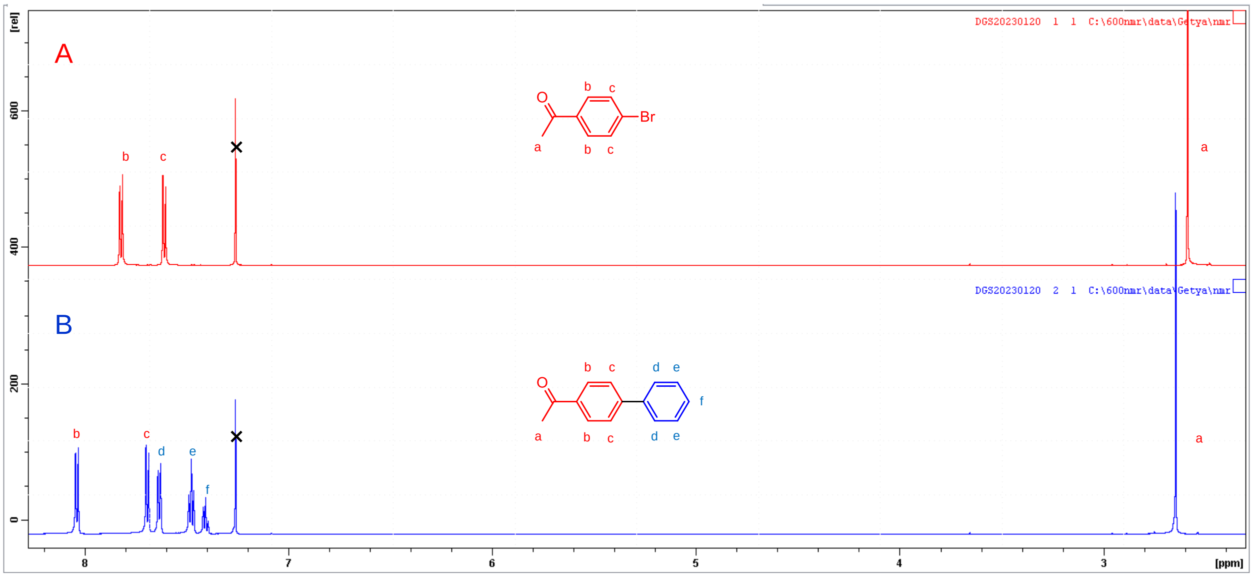

Supplement: Supplementary file 1 [file polymers-15-01671-s001.zip › polymers-2283986-supplementary.pdf]
